# Low-Complexity Channel Codes for Reliable Molecular Communication via Diffusion

**DOI:** 10.3390/s22010041

**Published:** 2021-12-22

**Authors:** Sofia Figueiredo, Nuno Souto, Francisco Cercas

**Affiliations:** 1 Telecommunications and Computer Engineering, Instituto Universitário de Lisboa (ISCTE-IUL), 1649-026 Lisboa, Portugal; sadfo@iscte-iul.pt (S.F.); nuno.souto@lx.it.pt (N.S.); 2 Instituto de Telecomunicacoes, 1049-001 Lisboa, Portugal

**Keywords:** future wireless networks, 6G, molecular communications, diffusion-based, channel coding, TCH codes

## Abstract

It is envisioned that healthcare systems of the future will be revolutionized with the development and integration of body-centric networks into future generations of communication systems, giving rise to the so-called “Internet of Bio-nano things”. Molecular communications (MC) emerge as the most promising way of transmitting information for in-body communications. One of the biggest challenges is how to minimize the effects of environmental noise and reduce the inter-symbol interference (ISI) which in an MC via diffusion scenario can be very high. To address this problem, channel coding is one of the most promising techniques. In this paper, we study the effects of different channel codes integrated into MC systems. We provide a study of Tomlinson, Cercas, Hughes (TCH) codes as a new attractive approach for the MC environment due to the codeword properties which enable simplified detection. Simulation results show that TCH codes are more effective for these scenarios when compared to other existing alternatives, without introducing too much complexity or processing power into the system. Furthermore, an experimental proof-of-concept macroscale test bed is described, which uses pH as the information carrier, and which demonstrates that the proposed TCH codes can improve the reliability in this type of communication channel.

## 1. Introduction

The telecommunications domain is constantly developing, facing all types of new demands, and therefore, while fifth-generation (5G) wireless networks are currently being deployed around the world, work is already underway for future evolutions such as sixth-generation (6G) systems [1] and even beyond. Extending the concept of Internet of Things [2] and emerging as a new network paradigm is the “Internet of Bio-Nano Things” (IoBNT), defined as the interconnection of nanoscale devices [3,4]. This is a revolutionizing concept promising many applications in the biomedical field, medicine, industry, environment, agriculture, and military [5]. Communications in IoBNT need to be biocompatible, energy-efficient, and robust in physiological conditions, making electromagnetic signals a difficult choice due to issues related to biocompatibility, power, and possible health hazards. An alternative to this problem is Molecular Communications (MC), using molecules for encoding, transmitting, and receiving information. MC are feasible, being considered easier to implement than other approaches in the near term. They are scalable having an appropriate size for nanomachines, and they are bio-compatible allowing the integration with living systems [6]. MC have been captivating a lot of research interest with several novel transmitter/receiver architectures being proposed in recent years. Due to the growing interest in nanoscale communications and MC, IEEE has already started standardization efforts through the IEEE P1906.1 working group [7,8]. Nevertheless, MC for in-body networks is still in its infancy, and it will take some time before practical systems become a reality, probably not before a seventh or higher generation (7G) of wireless networks [8]. However, MC are also relevant for several industry and environment applications such as smart infrastructure monitoring, quality control, hazardous environment monitoring, and plant and animal monitoring [9]. Many of these scenarios rely on macroscale MC which is expected to evolve much faster than in-body MC, and may even allow it to be integrated into the next generation of wireless networks, namely, into 6G [8].

Regarding this type of communications, diffusion-based MC is one of the most studied channels because of its flexibility and suitability for a wide number of scenarios. The diffusion-based architecture is characterized by the random movement of molecules (Brownian motion) from higher concentrations to less [10,11]. The system structure adopted for MC is similar to traditional radio communication systems, characterized by an end-to-end chain [12,13]: a transmitter, a channel, and a receiver. A few MC systems based on diffusion have been experimentally tested over the last few years. The purpose is to bridge the gap between theory and practice. Due to current technology limitations, most experiments are still carried out at the macro level instead of nano. For instance, an air-driven experiment was described in [14], which was based on spraying and detecting alcohol in open space. Some difficulties identified in this experiment were the fluctuations in humidity and temperature that change the channel features, which in the molecular context may have an impact on the results obtained. In a liquid-driven experiment presented in [15], the authors used small tubes as the communication channel where information is carried out via the pH. The authors refer that the system was shown to achieve better data rates than any previous chemical communication platform tested.

MC have some limitations: the stochasticity where molecules have random propagation called environmental noise, the large delays due to the much longer propagation times of molecules when compared to the speed of light, the range since known MC techniques have very short practical ranges, and the fragility because biological components can be environmentally sensitive [5,16]. These conditions pose many challenges for achieving a reliable communication. One of the main aspects when designing an MC system is the selection of the modulation which has a direct impact on the behavior of the channel and in the implementation complexity. In the case of MC, where molecules are the information carriers, several different schemes have been proposed [6,13,17]. These comprise approaches where information can be encoded via the emission of a single molecule type (e.g., with on–off keying (OOK)), changing the concentration (e.g., concentration shift keying (CSK)), type of molecules (e.g., molecule shift keying (MoSK)), ratio (e.g., isomer-based ratio shift keying (IRSK)), release time (e.g., release time shift keying (RTSK)), release order of molecules (e.g., molecular array-based communication (MARC)), as well as the base sequences of nucleotides (e.g., nucleotide shift keying (NSK)). Another aspect that plays a central role in the performance of MC communications is the detection task. Optimal and suboptimal detection methods have been proposed in the literature as addressed in [18], with some recent innovative approaches as in [19]. However, the complexity of many of the approaches can be excessive particularly, when one considers the need for instantaneous channel state information which, in the MC channel can be very challenging due to the fast time varying nature. To cope with this problem, non-coherent detection techniques can be adopted. This is the approach followed in [20], which relied on the comparison of the number of molecules in each interval against an adaptive threshold. While being simple, this detection scheme tends to perform poorly when sequences of consecutive ones occur. Another relevant component for reducing the effect of extreme noise and inter-symbol interference (ISI) that is typical in MC channels corresponds to channel codes. This is achieved by adding redundancy to the emitted message, and by making it possible to correct the errors caused by noise during transmission at the receiver, improving the reliability of the communication link. Some studies have been made, for instance, in [21,22] where authors demonstrated that Hamming codes are able to improve the performance of MC systems. However it was also shown that there is a Hamming order at which they become self-defeating, making it important to study other codes. Another proposed solution relies on the “ISI-mitigating channel codes” (ISI-mtg) [14], which is a code that has properties specially adapted to the MC channel environment, namely, the codewords do not contain consecutive ‘1’s, they always start with ‘0’, and they contain at least one bit ‘1’. This code proved to present a significant improvement in terms of bit error rate (BER).

Motivated by the work above, in this paper, we study Tomlinson, Cercas, Hughes (TCH) codes, as a new concept applied to MC systems. These codes possess several properties that make them appealing for various applications, such as error correction, synchronism, spread spectrum systems, and channel estimation [23,24,25]. The main goal of adopting these codes for MC is to achieve good coding gains due to a smaller autocorrelation distribution, that is, higher minimum distances, while supporting low implementation complexity based on soft-decision decoding for best performance. The main contributions of this paper can be summarized as follows:

- We introduced a new low complexity coding scheme, the TCH codes, targeted to the typically tightly constrained MC applications.
- We propose adequate symbol detection schemes for the MC channel which enable low-complexity hard and soft decision TCH decoding.
- The efficiency of the TCH encoder and decoder is analyzed and compared with other existing alternatives proposed in the literature. A thorough performance assessment of the resulting scheme is accomplished through an end-to-end MC simulator incorporating a diffusion-based channel model. Different pulse types and modulations are considered in this evaluation.
- We also present a macro-scale MC experiment, using pH as the information carrier combined with Manchester pulses, that was implemented for this study. This experiment aimed to validate the reliability of the TCH codes in an MC environment.

This paper is organized as follows. Section 2 describes the system model and the characteristics of the TCH transmitter and receiver. Section 3 presents the results of the simulated and experimental system. Finally, Section 4 summarizes the main conclusions of the paper.

## 2. Materials and Methods

### 2.1. Diffusion-Based Channel

Let us consider a diffusion-based MC system operating in a three-dimensional space. The model assumed for the average number of molecules counted at the receiver for this work is the point transmitter to a spherical absorbing receiver [26,27,28,29]. This type of receiver counts the number of absorbed molecules during an observation window $\left[t_{l},t_{u}\right]$. The probability, $F_{hit}\left(t_{u},t_{l}\right)$, of a molecule emitted at time *t* = 0 to be observed at the receiver during interval [$t_{l}$, $t_{u}$], can be defined as [26]

(1) $$F_{hit}\left(t_{u},t_{l}\right)=\frac{a_{rx}}{d_{0}}\left[erfc\left(\frac{d_{0}-a_{rx}}{\sqrt{4Dt_{u}}}\right)-erfc\left(\frac{d_{0}-a_{rx}}{\sqrt{4Dt_{l}}}\right)\right]$$

where erfc is the complementary error function, $a_{rx}$ is the radius of the spherical receiver and $d_{0}$ is the distance between the center of the transmitter and the center of the receiver. *D* is the diffusion coefficient of the ith molecule, m${}^{2}$s${}^{-1}$, which can be calculated using

(2) $$D=\frac{K_{B}T}{6\pi \eta R_{p}}$$

where $K_{B}=1.38\times 10^{-23}$ is the constant of Boltzmann, *T* is the temperature in Kelvin, η is the fluid viscosity, and $R_{p}$ is the radius of the particle.

The approach taken in this work assumes a diffusion-based MC system whose information bits are encoded in order to reduce ISI. The concentration vector corrupted by noise at the kth time interval can be written as

(3) $$y\left[k\right]=\sum_{l=0}^{L-1}h\left[l,k\right]x\left[k-l\right]+n\left[k\right]$$

where *L* is the assumed length of the ISI, $x\left[k\right]\in \left\{A_{0},A_{1}\right\}$ (with $A_{0}$ and $A_{1}$ representing the levels for a ‘0’ and a ‘1’) denotes a concentration based binary modulation symbol emitted from the transmitter at time *k*, h[l,k] is the number of molecules observed by the absorbing receiver during time interval *k* after $N_{molec}$ are released from the transmitter at time k−l and n[k] is the number of noise molecules detected by the receiver at time *k*. Both h[l,k] and n[k] are modeled as Poisson random variables: $h\left[l,k\right]∼Poisson(\bar{h}\left[l,k\right])$ and $n\left[k\right]∼Poisson\left(\bar{n}\right)$. Taking into account the probability model defined in (1), we have

(4) $$\bar{h}\left[l,k\right]=N_{molec}.F_{hit}\left(\left(l+1\right)T_{s},l.T_{s}\right).$$

**Figure 1:**
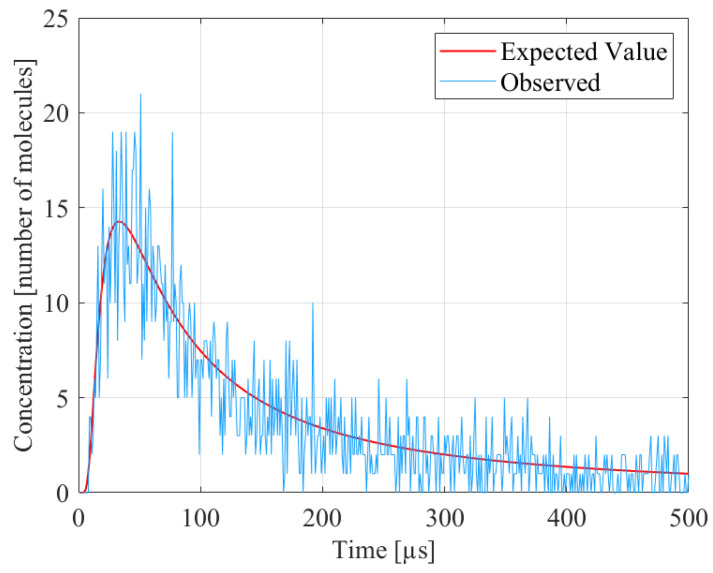
Example illustrating the expected and observed concentrations for a diffusion-based MC channel, assuming number of molecules ($N_{molec}$) = 10,000, *d* = 0.3 μm, *D* = 450 μm${}^{2}$/s, and $a_{rx}$ = 50 nm.

It can be shown that the received concentration at the receiver can be expressed as

(5) $$y\left[k\right]=\underset{\mathrm{desired}\;\mathrm{signal}}{\underset{︸}{\bar{h}\left[0,k\right]x\left[k\right]}}+\underset{\mathrm{diffusion}\;\mathrm{noise}}{\underset{︸}{v[k]}}+\underset{\mathrm{ISI}}{\underset{︸}{I[k]}}+\underset{\mathrm{environmental}\;\mathrm{noise}}{\underset{︸}{n[k]}}$$

where the diffusion noise follows the distribution $v\left[k\right]∼Poiss_{0}\left(\bar{h}\left[0,k\right]x\left[k\right]\right)$, i.e., it is equivalent to a Poisson random variable whose mean has been subtracted. This noise is signal dependent and accounts for the deviation of the concentration from its expected value according to the diffusion model, as illustrated in Figure 1.

### 2.2. Types of Pulses

**Figure 2:**
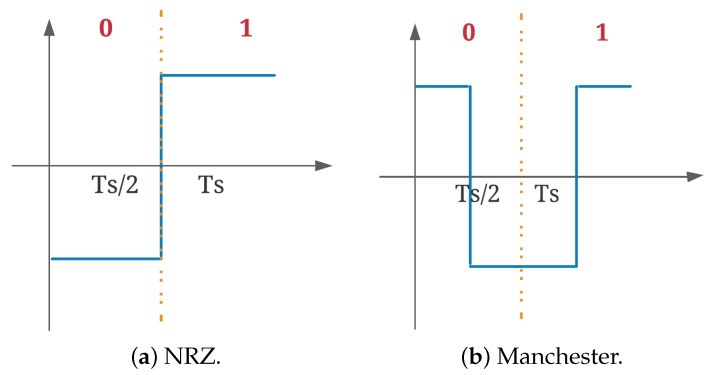
Type of pulses.

For the proposed MC system, in addition to the usual non-return to zero (NRZ) pulses exemplified in Figure 2a, Manchester pulses are also considered. These types of pulses, as in Figure 2b, have their wave boundaries always between 1 and −1; therefore, the decisions are usually taken in the middle of each bit [30]. These features will provide more robustness to the MC communication system in the experimental implementation.

### 2.3. Modulation

In MC, to convey distinct messages, each possible message is associated with a distinct molecule signal. To accomplished this, as defined in [17], the information can be modulated based on the properties of the molecules which allows different types of MC schemes: Concentration-based, Type-based, Timing-based, Spatial, or a combination of these (hybrid).

The concentration-based techniques are a representation of the information that relies on varying the concentration of the molecules. This is achieved by making the amount of released molecules carry information over discrete period time slots, the symbol slots. The simplest form of this approach, analogous to traditional communications, is the on–off keying (OOK), where each symbol represents a one-bit value. Bit-1 corresponds to the transmitter releasing a fixed number of molecules and bit-0 corresponds to the transmitter not releasing anything within the symbol slot. Another possible concentration-based technique is the Binary Concentration Shift Keying (BCSK) where in each symbol slot, sending a certain concentration of a molecule represents bit-1 and a different concentration represents bit-0. In this work, we will consider both OOK and BCSK.

### 2.4. Detection and Bit Decision

In this paper, we assume that no channel state information (CSI) exists at the transmitter nor the receiver. Therefore, the detection is accomplished solely based on the received observations over each encoded block duration. Two simple detection approaches are considered. The first one is a threshold based detection similar to that in [14], which generates hard decision symbols/bits. We consider a concentration-based binary modulation where each symbol comprising codeword *i* is represented as $x^{i}\left[k\right]\in \left\{A_{0},A_{1}\right\}$, with $A_{0}$ and $A_{1}$ denoting the levels for a ‘0’ and a ‘1’, respectively. In this case, an adaptive threshold is computed for the *i*-th codeword as

(6) $$t^{i}=ay_{min}^{i}+\left(1-a\right)y_{max}^{i}$$

where $y_{min}^{i}=min\left(y^{i}\right)$ and $y_{max}^{i}$ = max($y^{i})$, with $y^{i}={\left[y^{i}\left[1\right],\ldots ,y^{i}\left[N\right]\right]}^{T}$ denoting the vector with the corresponding *N* received samples. Parameter *a* is a scaling factor which, considering the adoption of a balanced code with equal number of ‘0’s and ‘1’s, we set as *a* = 0.5. Using this threshold, a hard decision estimate is simply obtained as

(7) $${\hat{x}}^{i}\left[k\right]=\left\{\begin{array}{cc} A_{0}, & \;y^{i}\left[k\right]<t^{i}\\ A_{1}, & \;y^{i}\left[k\right]<t^{i} \end{array}\right.$$

which can be directly demodulated into bits. The second detection technique relies on an ad hoc computation of log-likelihood ratios (LLRs) for each bit. As we assume that no prior knowledge about the statistics of the channel exists, we compute estimates for the probabilities $p_{1,k}=P\left(x^{i}\left[k\right]=A_{1}|y^{i}\right)$ and $p_{0,k}=P\left(x^{i}\left[k\right]=A_{0}|y^{i}\right)$ considering a uniform distribution whose bounds are defined by $y_{min}^{i}$ and $y_{max}^{i}$. In this case, these probabilities are obtained as

(8) $$p_{1,k}=\frac{y^{i}\left[k\right]-y_{min}^{i}}{y_{max}^{i}-y_{min}^{i}}$$

and

(9) $$p_{0,k}=\frac{y_{max}^{i}-y^{i}\left[k\right]}{y_{max}^{i}-y_{min}^{i}}.$$

Soft values can then be computed as the conditional expected value of the symbols which can be written as

(10) $${\tilde{x}}^{i}\left[k\right]=E\left(x^{i}\left[k\right]|y^{i}\right)=\left(A_{1}-A_{0}\right)p_{1,k}+A_{0}.$$

Taking into account that the LLRs for the individual bits can be expressed as

(11) $$\lambda^{i}\left[k\right]=log\left(\frac{p_{1,k}}{p_{0,k}}\right)$$

we can write

(12) $$p_{1,k}=\frac{1}{1+e^{-\lambda^{i}\left[k\right]}}$$

which allows us to compute the soft-values as

(13) $${\hat{x}}^{i}\left[k\right]=\frac{\left(A_{1}-A_{0}\right)}{1+e^{-\lambda^{i}\left[k\right]}}+A_{0}.$$

Note that in the case of OOK, with $x^{i}\left[k\right]\in \left\{0,A\right\}$, this expression can be rewritten as

(14) $${\hat{x}}^{i}\left[k\right]=\frac{A}{2}\left(\tan h\left(\frac{\lambda^{i}\left[k\right]}{2}\right)+1\right).$$

In the case of a polar modulation, where $x^{i}\left[k\right]\in \left\{-A,A\right\}$, (13) reduces to

(15) $${\hat{x}}^{i}\left[k\right]=A\left(\tan h\left(\frac{\lambda^{i}\left[k\right]}{2}\right)\right).$$

These soft estimates then allow simple soft-decision decoding based on squared Euclidean distance minimization.

While the assumption of uniform distribution used for computing (8) and (9) can be considered empirical, the simulation results presented in Section 3.1 show that it is in fact reasonable as the performances of TCH codes with this type of detection clearly outperform not only the uncoded case but also the same TCH codes using the hard decision approach. Furthermore, note that all the derived expressions for the computation of the LLRs do not depend on the specific distribution assumed for probabilities (8) and (9), which means that if a different distribution was to be considered (for example, due to some channel knowledge acquired by the receiver) only the computations in (8) and (9) would become different.

### 2.5. TCH Codes

Channel coding is based on correcting up to several errors, by providing immunity to the transmitted signal. Channel coding improves the communication link’s reliability, but it also implies a reduction in the overall transmission rate, due to implicit code rate k/n. TCH codes are a class of binary, nonlinear, non-systematic, and cyclic block codes of length $n=2^{m}$, where *m* is any positive integer. For efficiency reasons, the all-zero codeword was excluded from the code set and the inverse (binary negation) of any codeword is always another valid codeword [23]. TCH block codes are identified as TCH(n,k,t) where *n* represents the code length, *k* identifies the number of information bits in a code word and *t* is its error-correcting capacity. With $P_{i}$ being a theoretically generated set of Base Polynomials, which give the whole structure for any of these codes, we can write [24]

(16) $$n=2^{m},m\in N$$

(17) $$k=m+\log_{2}\left(h\right)+1$$

(18) $$d_{min}^{TCH}\geq 2t_{TCH}+1$$

(19) $$d_{min}^{TCH}\leq H_{d}\left[P_{i}\left(x\right),\left\{P_{j}\left(x^{r}\right)\right\}\mathrm{mod}\;n_{TCH}\right]\leq n_{TCH}-d_{min}^{TCH}$$

(20) $$P_{i}\left(x\right)=P_{j}\left(x^{r}\right)\mathrm{mod}\;n_{TCH},i\neq j\forall t_{TCH}\in N.$$

As usual, their error-correcting capacity, $t_{TCH}$, depends on the minimum distance, $d_{min}^{TCH}$, between the polynomials, where $H_{d}$ stands for the Hamming distance between any two polynomials. TCH codes are also balanced codes, that is, the number of zeros equals the number of ones in each codeword, which is an important feature for MC systems. We can derive TCH codes of nearly any length, however, the most important class of TCH codes which originated all of them, are based on the so-called Basic TCH Polynomials (B-TCH). B-TCH polynomials with degree *n* are obtained by the following equation

(21) $$P\left(x\right)=\sum_{i=0}^{\frac{p-1}{2}-1}a_{i}x^{K_{i}},a^{i}\in GF\left(q\right),q=p^{k},k\in N$$

where the exponents $K_{i}$ satisfy the equation

(22) $$a^{K_{i}}=1+a^{2i+1},i=0,1,\cdots ,\frac{p-1}{2}-1.$$

In these expressions, *p* is a prime number obeying the following condition:

(23) $$p=n_{TCH}+1=2^{m}+1.$$

**Figure 3:**
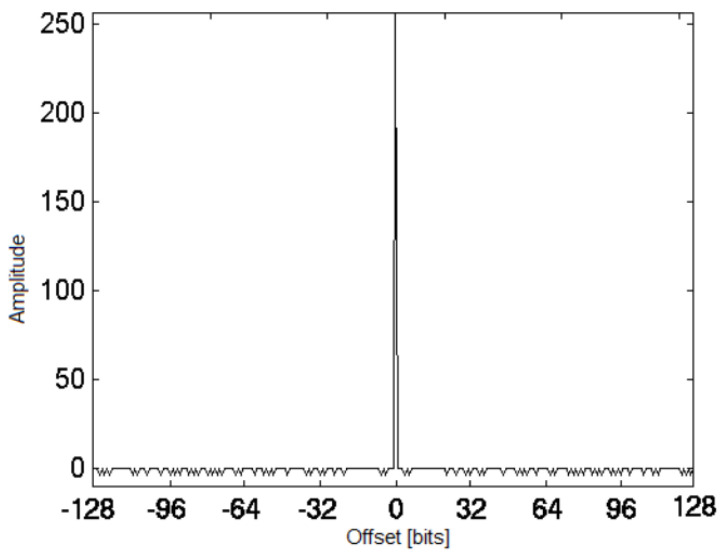
Typical autocorrelation function of a B-TCH polynomial with *n* = 256, [24].

Prime number that verify this condition can be written as

(24) $$F_{i}=2^{2^{i}}+1,i\in N$$

and so they are in fact Fermat numbers ($F_{i}$). Only five code lengths obey these rules, which means that we can only generate pure TCH polynomials for codes with lengths of 2, 4, 16, 256, and 65,536. TCH codes originated by B-TCH polynomials have both good cross and autocorrelation. Their autocorrelation is always three-valued, with values *n*, 0, and –4, and its distribution is perfectly characterized for any *n* [25]. This translates into a great advantage for higher sized TCH codes, such as TCH codes length *n*≥ 256 since these sequences tend to get closer to a Dirac impulse, as shown in Figure 3.

**Figure 4:**
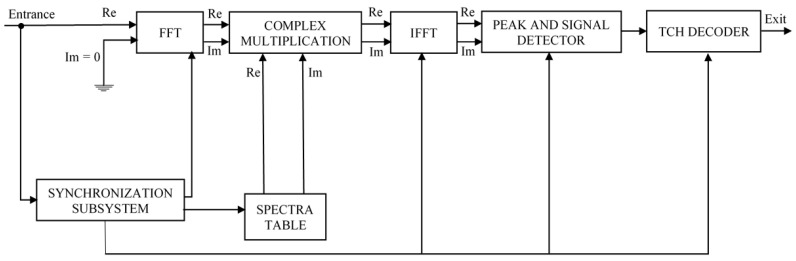
TCH decoder [25].

Conceptually, the TCH receiver is based on a group of correlators to compare the received word. As usual, and by comparing the outputs of the correlators it becomes possible to choose the sequence that corresponds to the highest correlation or the most likely word sent. In the TCH receiver, depicted in Figure 4, correlation is done in the frequency domain using two FFT’s, a complex multiplication and an IFFT, which, together with some optimizations, accelerates the decoding process. For example, and as FFT’s of length *n* process *n* codewords simultaneously, the efficiency increases with the code length *n*. Furthermore since both, real and imaginary parts are also processed simultaneously, that further decreases the processing time to half, again doubling the receiver efficiency.

### 2.6. Experimental Setup

To help validate the performance of the proposed TCH-based MC communications, we implemented a macroscale test bed. In our experiment, we used pH levels to test the molecules as information carriers. The different pH measurements are indicators of how acidic or basic the water is: pH’s above 7 indicate a basic solution and below 7 an acidic solution, on a scale between 0 and 14. The pH measures the amount of free hydrogen (H${}^{+}$) and hydroxyl (OH${}^{-}$) in the water. This means that water with more free hydrogen ions is acidic and water with more free hydroxyl ions is basic. The scale on which pH is measured is logarithmic, which means that increasing or decreasing an integer value changes the concentration by a factor of ten [31,32]. Thus, we use an acid and a base to send information. The used base solution has a pH level of 9 and the used acid solution has a pH of 3. Utilizing pH could constitute a problem because of the limited scale, from 0 to 14. For example, in the case of bit 1 being represented by the base and the bit-0 by the acid, if many consecutive bit-1’s were transmitted, information could be lost. To avoid this, we decided to use Manchester codes in this experiment to transmit the bits. Thus, bit-1 is represented by sending an acid and then a base, and bit-0 is represented by sending a base and then an acid. This prevents the information from being lost as it ensures the same amount of acid and base solution is released in the channel independently of the information sequence.

**Figure 5:**
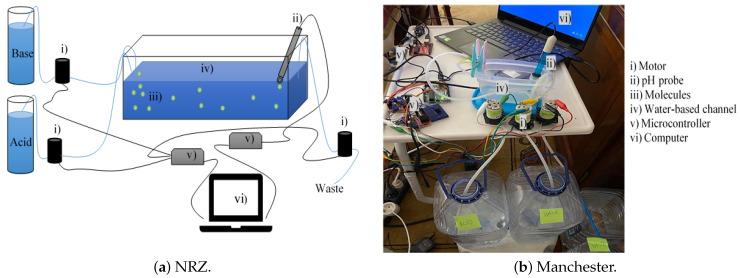
(**a**) Schematic diagram of the experimental implementation. (**b**) Photo of the experiment.

Next, we will explain the constituents of the system namely, the transmitter, the channel, and the receiver. The main diagram of the experiment is presented in Figure 5a.

#### 2.6.1. Transmitter

The transmitter is composed of two containers: one for the basic solution and another for the acid solution. These containers with the solutions are connected through a silicone tube to peristaltic pumps. Because we have an acidic and a basic solution, we used two peristaltic pumps to transmit the solutions individually and avoid contamination. There is also a third pump that removes the liquid, working at the same speed as the other two pumps mentioned above. These pumps feature a flexible tube, giving an open flow path for the liquids to pass. The pumps we chose were 12 V aquarium pumps working at 5000 RPM. As these pumps work with a high current, a driver is used. We control when they are on or off and how fast they run by using a microcontroller (a simple Arduino UNO microcontroller was adopted) connected to a computer, as in Figure 5. According to the transmitted information, a certain quantity of an acid solution or a basic solution is released to the channel.

#### 2.6.2. Channel

As represented in Figure 5, we use a container to simulate the channel. We decided to fill the container with only 20 mL of water with a pH of approximately 7. On one side of the container, we placed the tubes sending the acid or basic solution, one at a time (never simultaneously). On the other side of the container, 6 cm apart, we placed the pH probe and another pump to keep the container’s liquid volume always the same. Although these pumps generate some flow, the system will still mainly work via diffusion. One advantage of using acids and bases is that they are chemicals that cancel each other, and that property is very important in closed loops because the average concentration of these chemicals would remain constant assuming the same transmission concentration of bases and acids.

#### 2.6.3. Receiver

The receiver uses a different microcontroller, also an Arduino Uno, connected to the main computer. This board function is to read the pH levels in the water through the pH probe, as shown in Figure 5. We used a 5 V pH probe, featuring, as most pH probes, a glass bulb filled with strong electrolytes and a silver wire inside. This allows the pH electrode to measure the difference in potentials between the two sides of the glass electrode. This signal is then converted into an electric potential which allows reading the pH value. The pH probe is connected to a pH meter, which is connected to the Arduino Uno microcontroller, that digitizes the pH reading through a 10-bit analog to digital converter (ADC). This probe can introduce a pH measurements error of ±0.1 at 25 ℃.

## 3. Results and Discussion

### 3.1. Simulation Results

Due to the fact that in-body MC technology is still in its infancy, analysis of MC systems typically has to be made resorting to numerical evaluation using end-to-end MC channel models. Note that this type of numerical evaluation is very important not only to understand the possible behavior of a scheme, but also as it allows us to capture the potential achievable gains between different solutions. Therefore, in this section we perform a thorough assessment of the proposed scheme using an end-to-end MC simulator that integrates a diffusion-based MC channel model. The purpose of this simulation is to test different modulations and bit encoding solutions combined with TCH channel codes. We will be comparing the performance of some previously studied codes for MC against the TCH codes which we propose here as a potential alternative for MC scenarios. For the channel correcting codes, we choose to test the Hamming Codes (HC) because they are basic and easy to implement, and the ISI-mtg codes from [14] because they were designed specifically for an MC environment. Finally, we test the TCH codes for different codes lengths. To guarantee a fair comparison between these codes, similar sets of code rates were used comprising both lower codes rates, and higher codes rates. We remind that lower coding rates may obtain better performances but decrease the information bit rate. Higher coding rates can improve the bit rate sacrificing some BER performance. For the uncoded scenario and for the HC we used threshold hard decision. For the TCH codes and for the ISI-mtg codes, LLR soft decisions were employed. Both these bit decisions are detailed in Section 2.4.

The main parameters used in the simulations are described in Table 1. The symbol duration can have a critical impact on the behavior of the system. To evaluate the impact of this parameter we will consider different values for the scaling factor τ which is used for defining the symbol duration, as shown in Table 1. Tests will be performed for a small symbol duration because it is when there are more errors, becoming very noticeable how channel codes help to correct them. The simulations were performed using NRZ and Manchester pulses, at different symbol durations, with two distinct types of modulation: OOK and BCSK. In the case of OOK modulation, we have x[k]∈{0,1}, whereas in the BCSK we use x[k]∈{−1,1}. For the BCSK we assume the existence of a molecules destructor that can reduce the concentration of the carrier molecule.

It is important to emphasize that while the channel model may not be the most adequate for this particular scenario, the proposed scheme does not depend on a specific channel and does not require channel knowledge. Therefore, using this channel model still enables an evaluation of the system behavior. The results will be presented as the relation between the BER and $N_{molec}/\sigma_{n}^{2}$ (per information bit) in dB, where $\sigma_{n}^{2}$ denotes the variance of the environmental noise.

**Figure 6:**
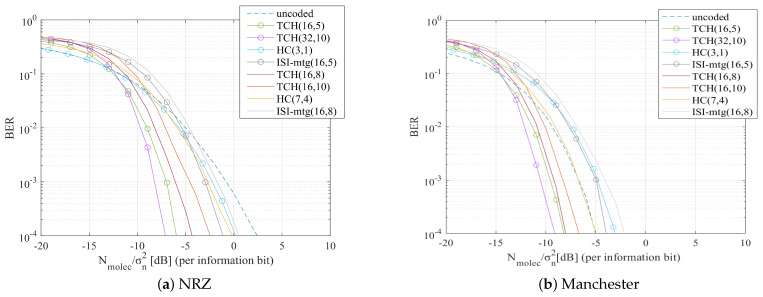
BER comparison for all the considered channel codes, using BCSK modulation and with τ = 2.

**Figure 7:**
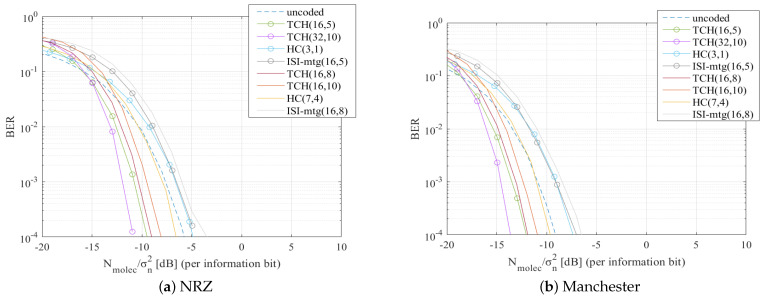
BER comparison for all the considered channel codes, using BCSK modulation and with τ = 10.

In Figure 6a and Figure 7a, we present the results for when NRZ pulses were tested, whereas in Figure 6b and Figure 7b, we present the results for when Manchester pulses were tested. Both Figure 6 and Figure 7 were simulated using BCSK modulation. When Manchester codes were used, the tested channel coding methods had an overall better performance. The big difference between these figures relies on the τ value, which in Figure 6 is 2, while in Figure 7 is increased to 10. Analyzing the results for the different τ values, it is noticeable a BER improvement in Figure 7 which has a higher τ value, and thus a higher $T_{s}$. Increasing the $T_{s}$ means that the molecules will have more time to diffuse and reach the receiver, reducing the ISI. Note, however, that a higher $T_{s}$ also means a transmission rate decrease.

**Figure 8:**
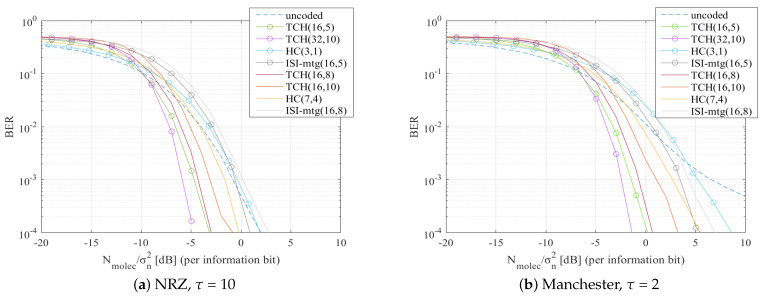
BER comparison for all the considered channel codes, using OOK modulation.

We tested the system with the OOK modulation, see Figure 8. In Figure 8a are the results for NRZ pulses with τ = 10, and in Figure 8b are the results for Manchester pulses at τ = 2. Comparing as a first case Figure 7a with Figure 8a, and as a second case Figure 6a with Figure 8b, the results show that the tested channel codes methods had worse BER results in Figure 8, when OOK modulation was used. In the first case, considering a BER reference of $10^{-2}$, it is observed an overall improvement of approximately 5 dB in terms of $N_{molec}/\sigma_{n}^{2}$ when the BCSK modulation is used. For the same reference BER of $10^{-2}$ it can be observed in the second case (comparison between Figure 6b and Figure 8b), an improvement of approximately 10 dB in terms of $N_{molec}/\sigma_{n}^{2}$ when BCSK modulation is used.

In Figure 6, Figure 7 and Figure 8 we can observe that TCH codes clearly had a lower BER than codes with similar rates:

- TCH(16,5) and TCH(32,10) codes performed better than HC(3,1) and ISI-mtg(16,5). For instance, in Figure 6b for a BER reference of $10^{-2}$ it can be observed a gain between 4 dB to 5 dB in terms of $N_{molec}/\sigma_{n}^{2}$;
- TCH(16,8) and TCH(16,10) codes performed better than HC(7,4) and ISI-mtg(16,8). This is illustrated, for example, in Figure 7a for a BER reference of $10^{-2}$, they had a gain between 1 dB to 2 dB compared with the HC(7,4) and a gain between 3 dB to 4 dB compared with the ISI-mtg(16,8).

Furthermore, TCH codes with higher codes rates had a better BER performance than other codes with lower rates:

- TCH(16,8) and TCH(16,10) were able to perform better than the HC(3,1) and the ISI-mtg(16,5). For example, in Figure 8a and considering a BER reference of $10^{-2}$, it can be observed a gain between 2 dB to 3 dB in terms of $N_{molec}/\sigma_{n}^{2}$.

As expected, the TCH codes with the same code rate but longer codewords, namely the TCH(32,10) when compared against the TCH(16,5), achieve better BER performances. For instance in Figure 6b the TCH(32,10), considering a reference BER of $10^{-2}$, had an improvement of approximately 1dB in terms of $N_{molec}/\sigma_{n}^{2}$ comparing to the TCH(16,5). Note that longer codewords incur in an additional delay. The results of our simulations proved that the TCH codes achieved a better BER performance against the other tested channel correcting codes, independently of the symbol duration, type of pulse, and modulation.

### 3.2. Experimental Results

To add validation to our simulation results, we performed a macro-scale MC experiment using TCH codes. The experimental setup did not intend to exactly mimic an in-body diffusion-based MC scenario, nor was the intention to repeat the same tests and comparisons that were already accomplished in the simulations. This evaluation was carried out by testing the channel without channel correcting codes and by testing the channel with a TCH(16,10) code. We chose to work with the TCH(16,10) code because it has a relative high coding rate while still maintaining good error capabilities. In this experiment we used Manchester pulses, for better channel robustness, combined with BCSK modulation. For transmitting a bit, we define two moments: a first moment that starts at the beginning of the symbol and goes to $T_{s}/2$, and a second moment that goes from $T_{s}/2$ to $T_{s}$. To transmit the bit-0, we start by sending a base solution during the beginning until halfway of the first moment, followed by a period until the end of the first moment when nothing is released to the channel. In the second moment, an acid solution is sent to the channel until halfway of the second moment followed by a period until the end of the symbol where nothing is released. To transmit bit-1, the opposite happens, in the first moment an acid followed by a period when nothing is released, and in the second moment a base followed by a period when nothing is released.

In our experiment, we collected data from 100 transmissions, where each transmission comprised a block with 100 random generated bits. To test how the symbol interval ($T_{s}$) could affect the received bit stream, we performed the experience for $T_{s}$ = 20 s and then for $T_{s}$ = 40 s. Is it important to underline due to the adopted experimental setup, there may exist other components contained in the chemicals used which can interfere with the channel characteristics and affect the pH probe reading. Still, the implemented communication scheme does not require channel state knowledge to accomplish the detection.

**Figure 9:**
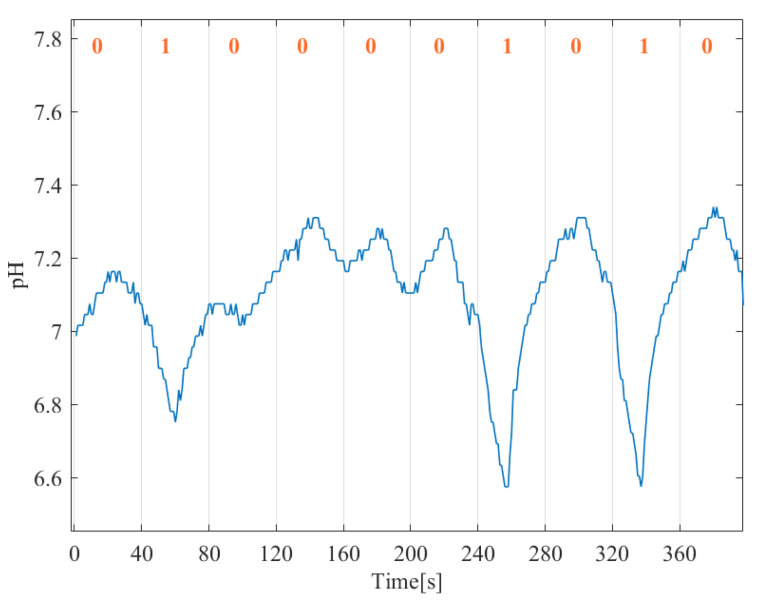
Example of 10 received bits with $T_{s}$ = 40 s after initial delay removal.

**Figure 10:**
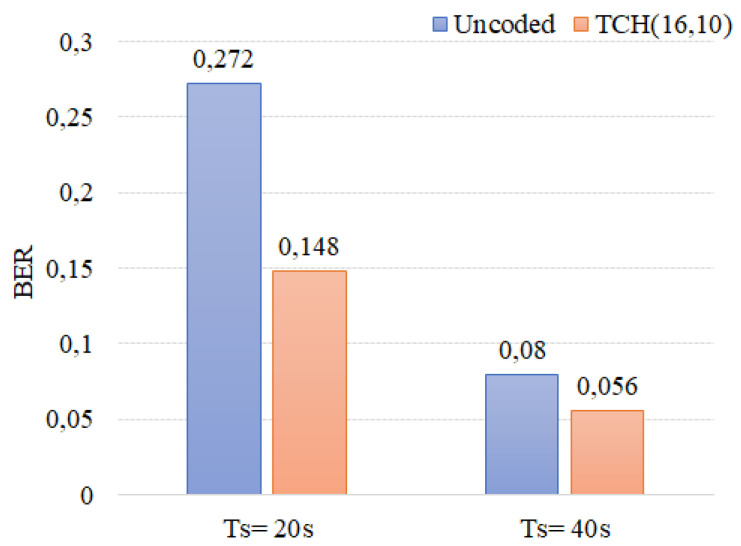
Uncoded and TCH(16,10) performance for $T_{s}$ = 20 s and $T_{s}$ = 40 s.

The first released molecules take time to reach the receiver (initial delay). In Figure 9 we show an example of the received bit stream after the initial delay removal. In Figure 10, it can be observed that when a larger $T_{s}$ is adopted the error rate decreases both in the uncoded and coded cases. This was also observed in the simulation results, Section 3.1, and was expected as, although affecting the transmission rate, a lower symbol time gives the system more time to receive most of the late molecules, thus decreasing the ISI. Furthermore, analyzing the results in Figure 10, it is clear that fewer errors were obtained when TCH codes were applied, confirming the TCH codes efficiency on correcting errors in an MC environment.

## 4. Conclusions

In this paper, we studied the adoption of TCH codes as a promising low-complexity solution for enabling reliable MC. Adequate detection and decoding methods which do not require CSI acquisition were presented and evaluated through end-to-end simulations. Furthermore, to validate the reliability achieved with the proposed approach, we implemented a macroscale MC platform where the channel is an aqueous solution and the information to be transmitted is differentiated by the pH value of the medium, as this is a good approach to a biological environment for in-body communications. At the transmitter, different encoded pulses were considered, namely, NRZ and Manchester, combined with either OOK and BCSK modulations. Other channel codes were evaluated as benchmarks for the TCH codes, namely, Hamming codes and the ISI-mtg codes that were designed specifically for this context.

The results obtained from the simulation implementation, showed that the new introduced coding scheme based on TCH codes, performed better without introducing more complexity to the system. In fact, TCH codes with higher rates were able to perform better than other codes with lower rates. In the developed macroscale experiment TCH codes proved to work when applied to a physical MC scenario. In addition, Manchester pulses for coded and uncoded situations proved to be essential when operating in a channel that can saturate, as in the case of the pH-based experimental tests. These pulses also proved to work better in the simulation than the NRZ pulses, giving more robustness to the communication in an MC channel with an absorbing receiver.

## Figures and Tables

**Table 1:** Simulation parameters.

Parameter	Value
Deterministic model	Point transmitter to a spherical absorbing receiver
Statistical model	Gaussian approximating Poisson
Paired tx−rx link distance ($d_{0}$)	10 (μm)
Diffusion Coefficient (*D*)	79.4 (μm${}^{2}$/s)
Transmitter radius and receiver radius ($a_{tx},a_{rx}$)	5 (μm)
Symbol duration ($T_{s}$)	$\tau \frac{{\left(d_{0}\right)}^{2}}{6D}$ (s)
